# Supplementary material for: A Non-Nutritive Feeding Intervention Alters the Expression of Efflux Transporters in the Gastrointestinal Tract
Source: Pharmaceutics. 2021 Oct 26;13(11):1789. doi: 10.3390/pharmaceutics13111789 (PMC8624582; doi:10.3390/pharmaceutics13111789)
Supplement: Supplementary file 1 [file pharmaceutics-13-01789-s001.zip › pharmaceutics-1399743-supplementary.pdf]

pharmaceutics

MDPI

Supplementary Materials: A Non-Nutritive Feeding Intervention Alters the Expression of Efflux Transporters in the Gastrointestinal Tract

Yang Mai, Francesca K.H. Gavins, Liu Dou, Jing Liu, Farhan Taherali, Manal E. Alkahtani, Sudaxshina Murdan, Abdul W. Basit and Mine Orlu

S1. Fibre meal description

The fibre meal was composed of cellulose. The fibre meal was provided as cellulose pellets (Figure 1A) where Solka Flocc 200 cellulose was pelleted. To obtain, the fibre meal suspension, the cellulose powdered were crushed using a pestle and mortar. The resulting powder is shown in Figure 1A. The powder was composed of cellulose fibres, off-white in colour and heterogeneous in particle size uniformity. Light microscope images of the powder is shown in Figure 1B and C.

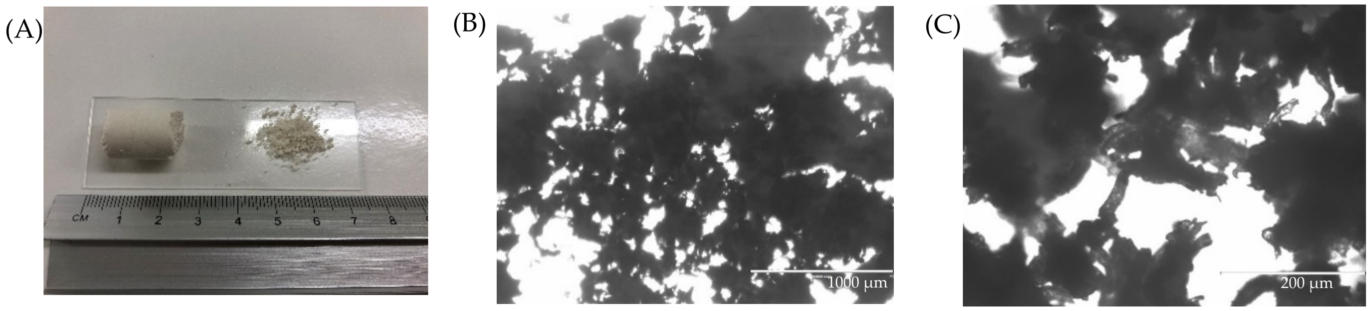

Figure S1. Images of (**A**) left cellulose pellet and right powdered cellulose, (**B**) and (**C**) light microscope images.

S2. Swelling capacity

The powders were assessed for swelling properties. The powder (0.5 g) was suspended in deionised water (4 mL) under magnetic stirring for 4 hours. Filter paper was used to remove water after 4 h. Equation 1 was used to calculate the swelling capacity of the cellulose powder, where W_s was the weight of the suspension and W_p was the weight of the powder.

$$Swelling\ capacity = \frac{W_s - W_p}{W_p} \quad (S1)$$

The cellulose particle has a swelling capacity of 160%. Figure S2 shows the fibre meal suspension.

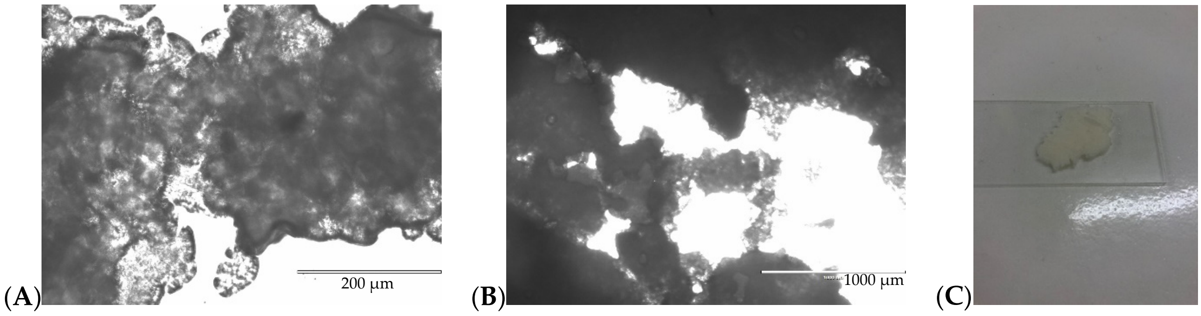

Figure S2. (**A**), (**B**) Light microscope images of fibre cellulose suspension and (**C**) image of fibre cellulose suspension after 4 h of magnetic stirring.

Table S1. P-glycoprotein (P-gp) expression (ng/mg) across the intestinal tract in male (blue) and female (pink) from time 0 to 2 h under fasted and fed states (normal meal and fibre meal (mean ± SD, n = 6).

**P-gp male fasted at time = 0h**		
	**Mean**	**SD**
**Duodenum**	9.89	0.42
**Jejunum**	12.42	0.70
**Ileum**	12.95	0.31
**P-gp male normal meal fed at time = 0.5 h**		
	**Mean**	**SD**
**Duodenum**	10.52	0.67
**Jejunum**	10.35	0.35
**Ileum**	10.71	0.49
**P-gp male normal meal fed at time = 1 h**		
	**Mean**	**SD**
**Duodenum**	10.65	0.46
**Jejunum**	8.58	0.23
**Ileum**	8.97	0.28
**P-gp male normal meal fed at time = 2 h**		
	**Mean**	**SD**
**Duodenum**	10.36	0.65
**Jejunum**	9.97	0.49
**Ileum**	10.24	0.54
**P-gp male fibre meal fed at time = 0.5 h**		
	**Mean**	**SD**
**Duodenum**	12.49	0.12
**Jejunum**	17.07	0.56
**Ileum**	16.81	0.37
**P-gp male fibre meal fed at time = 1 h**		
	**Mean**	**SD**
**Duodenum**	11.93	0.20
**Jejunum**	18.54	0.38
**Ileum**	18.16	0.40

**P-gp female fasted at time = 0h**		
	**Mean**	**SD**
**Duodenum**	10.67	0.70
**Jejunum**	10.58	0.35
**Ileum**	10.32	0.39
**P-gp female normal meal fed at time = 0.5 h**		
	**Mean**	**SD**
**Duodenum**	10.59	0.29
**Jejunum**	16.13	0.48
**Ileum**	10.86	0.48
**P-gp female normal meal fed at time = 1 h**		
	**Mean**	**SD**
**Duodenum**	10.75	0.25
**Jejunum**	17.30	1.32
**Ileum**	11.09	0.42
**P-gp female normal meal fed at time = 2 h**		
	**Mean**	**SD**
**Duodenum**	10.99	0.52
**Jejunum**	12.97	1.17
**Ileum**	10.77	0.23
**P-gp female fibre meal fed at time = 0.5 h**		
	**Mean**	**SD**
**Duodenum**	12.91	0.57
**Jejunum**	16.36	1.07
**Ileum**	15.71	0.40
**P-gp female fibre meal fed at time = 1 h**		
	**Mean**	**SD**
**Duodenum**	12.11	0.24
**Jejunum**	16.68	1.33
**Ileum**	16.04	0.34

P-gp male fibre meal fed at time = 2 h		
	**Mean**	**SD**
**Duodenum**	11.58	0.24
**Jejunum**	19.07	0.48
**Ileum**	15.84	0.44

P-gp female fibre meal fed at time = 2 h		
	**Mean**	**SD**
**Duodenum**	11.97	0.46
**Jejunum**	18.38	0.90
**Ileum**	17.02	0.33

Table S2. Breast cancer resistance protein (BCRP) expression (ng/mg) across the intestinal tract in male (blue) and female (pink) from time 0 to 2 h under fasted and fed states (normal meal and fibre meal) (mean ± SD, n = 6).

BCRP male fasted at time = 0h		
	**Mean**	**SD**
**Duodenum**	8.24	0.55
**Jejunum**	8.88	0.89
**Ileum**	9.87	0.24
**BCRP male normal meal fed at time = 0.5 h**		
	**Mean**	**SD**
**Duodenum**	8.54	0.41
**Jejunum**	9.50	0.28
**Ileum**	10.08	0.44
**BCRP male normal meal fed at time = 1 h**		
	**Mean**	**SD**
**Duodenum**	8.79	0.31
**Jejunum**	9.61	0.70
**Ileum**	10.59	0.68
**BCRP male normal meal fed at time = 2 h**		
	**Mean**	**SD**
**Duodenum**	9.04	0.36
**Jejunum**	11.37	1.02
**Ileum**	10.96	0.88
**BCRP male fibre meal fed at time = 0.5 h**		
	**Mean**	**SD**
**Duodenum**	10.00	0.48
**Jejunum**	13.21	2.28
**Ileum**	13.59	0.60
**BCRP male fibre meal fed at time = 1 h**		
	**Mean**	**SD**
**Duodenum**	11.15	0.38
**Jejunum**	13.25	0.65
**Ileum**	13.14	0.73
**BCRP male fibre meal fed at time = 2 h**		
	**Mean**	**SD**
**Duodenum**	12.82	0.54
**Jejunum**	14.22	2.20

BCRP female fasted at time = 0h		
	**Mean**	**SD**
**Duodenum**	9.32	0.36
**Jejunum**	8.92	0.55
**Ileum**	10.30	0.49
**BCRP female normal meal fed at time = 0.5 h**		
	**Mean**	**SD**
**Duodenum**	11.40	1.95
**Jejunum**	11.13	2.26
**Ileum**	10.18	0.22
**BCRP female normal meal fed at time = 1 h**		
	**Mean**	**SD**
**Duodenum**	10.13	0.89
**Jejunum**	10.60	0.81
**Ileum**	9.94	0.35
**BCRP female normal meal fed at time = 2 h**		
	**Mean**	**SD**
**Duodenum**	9.93	0.35
**Jejunum**	9.96	0.27
**Ileum**	10.63	0.76
**BCRP female fibre meal fed at time = 0.5 h**		
	**Mean**	**SD**
**Duodenum**	10.63	0.42
**Jejunum**	12.93	1.14
**Ileum**	13.48	0.75
**BCRP female fibre meal fed at time = 1 h**		
	**Mean**	**SD**
**Duodenum**	12.08	0.46
**Jejunum**	14.00	0.43
**Ileum**	13.97	0.50
**BCRP female fibre meal fed at time = 2 h**		
	**Mean**	**SD**
**Duodenum**	13.04	0.27
**Jejunum**	14.74	0.76

**Ileum**	14.38	0.69

**Ileum**	14.49	0.55

Table S3. Multidrug resistance protein 2 (MRP2) expression (ng/mg) across the intestinal tract in male (blue) and female (pink) from time 0 to 2 h under fasted and fed states (normal meal and fibre meal) (mean ± SD, n = 6).

	**MRP2 male fasted at time = 0h**	
	**Mean**	**SD**
**Duodenum**	2.90	0.37
**Jejunum**	3.64	0.31
**Ileum**	4.06	0.28
	**MRP2 male normal meal fed at time = 0.5 h**	
	**Mean**	**SD**
**Duodenum**	3.70	0.25
**Jejunum**	6.37	0.52
**Ileum**	5.86	0.37
	**MRP2 male normal meal fed at time = 1 h**	
	**Mean**	**SD**
**Duodenum**	4.20	0.13
**Jejunum**	5.51	0.85
**Ileum**	6.28	0.27
	**MRP2 male normal meal fed at time = 2 h**	
	**Mean**	**SD**
**Duodenum**	3.98	0.46
**Jejunum**	5.08	0.64
**Ileum**	6.00	0.70
	**MRP2 male fibre meal fed at time = 0.5 h**	
	**Mean**	**SD**
**Duodenum**	4.80	0.26
**Jejunum**	7.15	0.22
**Ileum**	6.13	0.26
	**MRP2 male fibre meal fed at time = 1 h**	
	**Mean**	**SD**
**Duodenum**	4.95	0.19
**Jejunum**	7.26	0.70
**Ileum**	7.19	0.35
	**MRP2 male fibre meal fed at time = 2 h**	
	**Mean**	**SD**
**Duodenum**	5.83	0.17
**Jejunum**	7.39	0.07
**Ileum**	7.21	0.33

**MRP2 female fasted at time = 0h**		
	**Mean**	**SD**
**Duodenum**	3.84	0.31
**Jejunum**	4.23	0.57
**Ileum**	5.08	0.21
**MRP2 female normal meal fed at time = 0.5 h**		
	**Mean**	**SD**
**Duodenum**	3.84	0.24
**Jejunum**	4.46	0.49
**Ileum**	4.80	0.27
**MRP2 female normal meal fed at time = 1 h**		
	**Mean**	**SD**
**Duodenum**	4.10	0.19
**Jejunum**	4.28	0.72
**Ileum**	5.28	0.27
**MRP2 female normal meal fed at time = 2 h**		
	**Mean**	**SD**
**Duodenum**	4.11	0.43
**Jejunum**	4.51	0.57
**Ileum**	5.19	0.36
**MRP2 female fibre meal fed at time = 0.5 h**		
	**Mean**	**SD**
**Duodenum**	5.13	0.39
**Jejunum**	6.75	0.43
**Ileum**	6.72	0.51
**MRP2 female fibre meal fed at time = 1 h**		
	**Mean**	**SD**
**Duodenum**	5.36	0.39
**Jejunum**	6.82	0.68
**Ileum**	7.27	0.09
**MRP2 female fibre meal fed at time = 2 h**		
	**Mean**	**SD**
**Duodenum**	6.05	0.16
**Jejunum**	7.33	0.18
**Ileum**	7.56	0.68
